# Detection of Transgenes in Local Maize Varieties of Small-Scale Farmers in Eastern Cape, South Africa

**DOI:** 10.1371/journal.pone.0116147

**Published:** 2014-12-31

**Authors:** Marianne Iversen, Idun M. Grønsberg, Johnnie van den Berg, Klara Fischer, Denis Worlanyo Aheto, Thomas Bøhn

**Affiliations:** 1 GenØk –Centre for Biosafety, Tromsø, Norway; 2 Unit for Environmental Sciences and Development, North-West University, Potchefstroom, South Africa; 3 Department of Urban and Rural Development, Swedish University of Agricultural Sciences, Uppsala, Sweden; 4 College of Agriculture and Natural Sciences, School of Biological Sciences, University of Cape Coast, Cape Coast, Ghana; 5 Faculty of Health Sciences, UIT The Arctic University of Norway, Tromsø, Norway; Henan Agricultural Univerisity, China

## Abstract

Small-scale subsistence farmers in South Africa have been introduced to genetically modified (GM) crops for more than a decade. Little is known about i) the extent of transgene introgression into locally recycled seed, ii) what short and long-term ecological and socioeconomic impacts such mixing of seeds might have, iii) how the farmers perceive GM crops, and iv) to what degree approval conditions are followed and controlled. This study conducted in the Eastern Cape, South Africa, aims primarily at addressing the first of these issues. We analysed for transgenes in 796 individual maize plants (leaves) and 20 seed batches collected in a village where GM insect resistant maize was previously promoted and grown as part of an governmental agricultural development program over a seven year period (2001–2008). Additionally, we surveyed the varieties of maize grown and the farmers’ practices of recycling and sharing of seed in the same community (26 farmers were interviewed). Recycling and sharing of seeds were common in the community and may contribute to spread and persistence of transgenes in maize on a local or regional level. By analysing DNA we found that the commonly used transgene promoter *p35s* occurred in one of the 796 leaf samples (0.0013%) and in five of the 20 seed samples (25%). Three of the 20 seed samples (15%) included herbicide tolerant maize (NK603) intentionally grown by the farmers from seed bought from local seed retailers or acquired through a currently running agricultural development program. The two remaining positive seed samples (10%) included genes for insect resistance (from MON810). In both cases the farmers were unaware of the transgenes present. In conclusion, we demonstrate that transgenes are mixed into seed storages of small-scale farming communities where recycling and sharing of seeds are common, i.e. spread beyond the control of the formal seed system.

## Introduction

Maize is a highly diverse crop, and has over thousands of years been under human selection for adaptation to various environments and agricultural practices [1], [2]. Among the modern maize varieties are both hybrids (high yielding F1 progeny of crosses between inbred lines) and improved open pollinated varieties (OPVs, where an assemblage of relatively uniform phenotypes are developed, but without the genetic homogeneity of hybrid varieties). In the mid-1990’s the first genetically modified (GM) maize hybrids were developed.

GM maize and cotton hybrids expressing insecticidal Cry proteins (Bt-toxins) were in 1997 the first GM plants to be approved in South Africa. By 2013 South Africa had 2.3 million hectares of GM crops under cultivation, of which the majority was maize (representing 78% of the GM crops under cultivation) [3]. This included insect resistant and herbicide tolerant maize hybrids as well as stacked events where the mentioned traits have been combined in a single hybrid. Most of the GM maize in South Africa is grown by commercial farmers. However, GM maize varieties have also been introduced to South African smallholders farming mainly for subsistence. In the attempts to increase the yield of this staple crop, and thereby improve food security, the South African government has funded several agricultural programs during the last decade. Some of these programs have introduced GM maize (both insect resistant and herbicide tolerant) to smallholders [4], [5]. International seed and agrochemical companies have been included in the design and implementation of these government programs, and they have arranged demonstration trials and farmer workshops where they have promoted GM maize to smallholders in the region [4]–[6].

The South African Genetically Modified Organism Act of 1997 (GMO Act) [7] and its subsidiary legislation is the main tool for regulating contained use, trial and commercial releases, but also the import and export, of GMO’s in South Africa. The GMO Act requires that permits are granted by the appropriate regulatory authorities for all activities involving a GMO. Usually the permits require certain conditions for the use of a particular GMO based on the risk assessment of that GMO. Permit holders are required to keep records of plant locations, educate and monitor farmers to make sure management strategies are adhered to, and to have surveillance and management plans to counteract resistance development (both in insects and in weeds). End-users purchasing GM seed are required to sign technology licencing agreements with the permit holders where they certify their compliance to the permit conditions. Under the GMO Act all users, including end-users, have a responsibility to avoid adverse impacts on the environment and animal or human health from the use of GMO’s, and can thus be held liable if damage occurs, or if permit conditions are breached. In addition to the South African GMO Act, patent legislation and laws regulating the plant breeder’s rights also regulate the use of GMO’s [5].

The current management practices implementing biosafety guidelines and maintaining the effectiveness of the GM traits are based on experiences from large-scale systems [4]. Also the research done on gene flow between GM and non-GM crops have drawn experience from large-scale, industrialized, agricultural settings (mainly focusing on cross-pollination) [8], [9]. Thus, currents knowledge may not be directly applicable in smallholder agricultural systems. Several features of the South African smallholder system makes appropriate management of the GM crops particularly challenging: fields of different farmers are close together, informal practices of seed recycling and sharing are more common, several different varieties of maize are commonly planted together, and the small fields make it comparatively more inconvenient to include buffer zones between non-GM and GM crops [4], [10]–[12].

While commercial farmers operate within a formal system for trading of seed (formally approved seed producers and seed retailers selling certified seed), many smallholders all over the world rely on informal seed systems where they develop, recycle and share seeds. Subsistence farmers commonly recycle maize for a range of reasons, such as tradition, food security and reducing input costs, and the fact that commercial varieties are often not adapted to their specific agroecological conditions [5], [13], [14]. Studies have shown that when GM-seeds enter smallholder communities, permit and licencing regulations are often not adhered to, i.e. contracts are not signed and management practices not followed up [5], [15]. This is most likely caused by a combination of lack of practical feasibility and incomplete and inaccurate information given to smallholders [4], [16], [17].

Few studies have investigated gene flow from GM maize into non-GM varieties in smallholder systems. The studies that have been published mainly present data from Mexico [18], [19]. However, Aheto et al. [20] modelled the potential for transgene diffusion in a West African context (Ghana) and concluded that transgenes would likely spread and persist in small-holder communities. This could potentially have severe consequences for the grain export sector. Other studies of smallholder communities in Zambia, have come to similar conclusions with regards to spread and persistence [10]–[12]. Recycling and sharing of seeds are important factors to take into account when studying gene flow. Based on a Mexican context these practices have shown the potential to spread transgenes faster and over a much larger area than pollen flow alone [19]. A similar vulnerability for transgene flow can be expected in smallholder communities in several African countries [12]. In general, the capacity of many African states for assessing potential risks posed by GM crops are limited, and public awareness, participation and information sharing may be additional limiting factors on the African continent [10], [11]. A key risk issue is whether it is possible to withdraw GM varieties in case unexpected and undesirable effects would occur [4], [20].

The potential implications of introducing GM crops into smallholder agriculture are diverse, including economic, sociological and environmental consequences. Depending on specific circumstances including type of crop, trait and when relevant, co-technology (such as specific herbicides to which the GM crop is resistant), GM crops can be of benefit to the farmer, through reducing yield loss or increasing yield stability [21], [22] or reducing environmental impacts through reduced use of pesticides [23], [24]. However, the adoption of GM crops in smallholder systems, depending on the specific regulations in each country, may further limit options to recycle and share seeds (such as plant breeder’s rights), likely increasing input costs of farming.

In 2001, a multinational seed and agrochemical company (Monsanto) arranged demonstration trials and held a workshop in the village investigated in this study, where they introduced stem borer resistant Bt-maize (MON810) to smallholders. When the village entered an agricultural program in 2003 (the Massive Food Production Program, MFPP), the chief of the village decided to purchase Bt-maize, the only commercial maize variety previously tested in his village [5]. Participating farmers were supplied with seed and fertilizer and supported to pay for tractor cultivation services. Farmers participating in the program also gave away Bt-seed to farmers that were formally not enrolled. Due to delays in seed deliveries and planting some farmers chose to save Bt-seed for more favourable planting conditions. As a result original Bt- seed was still planted in the village in 2008, two years after the agricultural program officially ended [4].

A more recent programme (run by Ntinga OR Tambo Development Agency, referred to here as the Ntinga project) has introduced herbicide tolerant maize (NK603, tolerant to the glyphosate herbicide Roundup) to the village, starting in the 2010/2011 growing season. This programme was still running at the time of field work.

This paper reports on i) the screening for transgenes in external fields, home gardens and local household seed holdings; and ii) the seed management practices of farmers, including sharing and recycling, in this small-scale farming system in Eastern Cape, South Africa, to address current knowledge gaps on potential mixing of transgenes into local seed stores.

To our knowledge, these are the first data that tests whether GM maize may have entered the informal seed system of South African smallholder farmers.

## Materials and Methods

**Figure 1:**
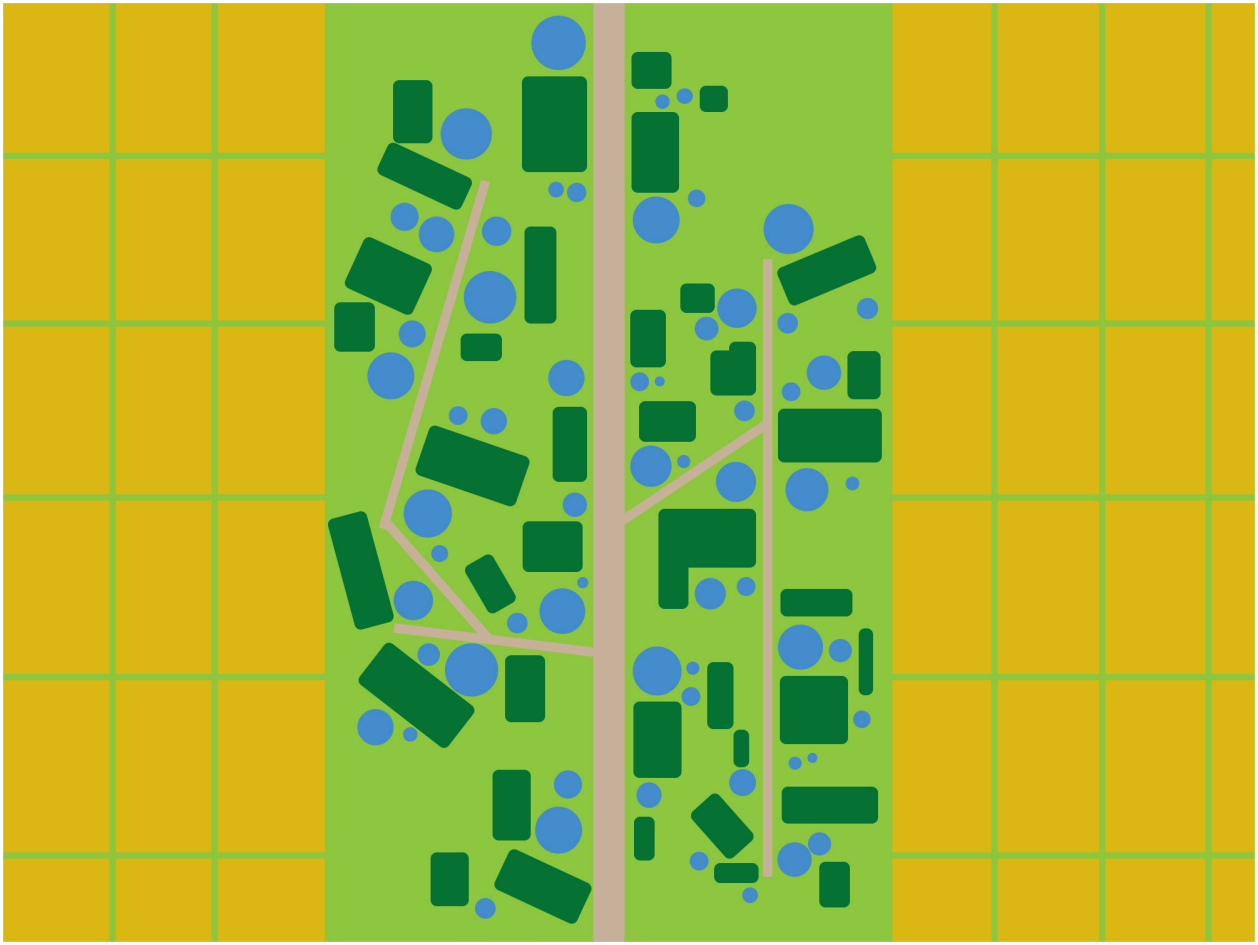
A South African Village Lay-Out. Drawing showing the layout of a South African smallholder village resembling the studied area with gardens (green) in connection to houses (blue) throughout the village itself, and larger area(s) where multiple fields with different owners lie close together (not to scale).

The material for this study, maize samples and farmer interviews, were collected over five days, in early December 2012, in a rural community in the north-eastern part of the Eastern Cape Province of South Africa. The study area has previously been described by Jacobson [5] and Jacobson and Myhr [4]. The sampling area consists of a nucleated village with communal grazing lands and fields surrounding the village. Each house has an adjacent home garden where maize is commonly planted together with other vegetables. Almost all houses have a field allocated in the communal field area outside the village. Fields are planted with maize, often intercropped with beans and pumpkins. An example of the layout of such a village is provided in Fig. 1.

DNA extractions and sample analysis was performed at the North-West University, Potchefstroom, South Africa, and at GenØk –Centre for Biosafety’s laboratory in Tromsø, Norway, respectively. GenØk’s laboratory is certified in accordance to Norwegian law for importing and working with GM material.

### Ethics statement

Prior to the study, an approval to conduct field work was sought from the traditional authority of the village. During each meeting with a farmer our team and the local assistants explained the research context to the farmer and asked for their willingness to participate. The assistants also explained to all participants that they could chose to decline from taking part in the study at any time. This included both the collection of seed and leaves from gardens and the questionnaire part. The mapping of fields were made based on consent from the local traditional authority as it was practically impossible during the time frame of this field work to connect individual farmers with individual fields and ask each farmer for consent. We however made sure to talk about what we were doing in the villages to avoid misunderstandings about the purpose of mapping the fields. Each farmer taking part in the data collection was given a number and we have avoided to mention farmer names and the name of the village and exact location.

### Maize leaf and grain sampling

Leaf samples were collected from 15 fields and 14 gardens. The fields that were sampled were randomly selected among the fields that were germinated, though fields with small seedling (one-leaf stage) were avoided. Gardens were selected based on consent from owners and the fields sampled were a subset of the fields that were planted. Thus the selection of fields and gardens was not randomized. Within fields and gardens sampling of leaves was randomized. Each sample set consisted of leaves from 30 different plants (one leaf per plant, 30 plants per field/garden), collected in a zigzag diagonal pattern inside the field or garden. A total of 892 leaves were collected, out of which 796 were used for transgene detection (due to varying DNA quality, see below). Since maize in gardens was planted earlier in the season than in fields, leaves collected from gardens were generally larger (plants around 0.5–1 meter tall) than leaves collected from plants in the fields which were at the two- or three-leaf stage upon sampling. When collecting samples from gardens, farmers were asked about the variety and the origin of seeds that they had used (i.e. any varietal names used by the farmer, and if the seed was recycled, store bought or a gift from a friend or relative) (see S1 Table for further information on the garden samples). Such information was not available for the samples collected in the fields.

Leaf samples were stored in paper envelopes at ambient temperature for four days until arrival in the laboratory.

A total of 20 seed samples (each consisting of a handful of seeds, approximately 99±17 seeds) was donated by 16 different farmers (some farmers had several varieties). Information was collected on the variety and origin of the seeds as well (see S2 Table for further information on the seed samples).

### Farmer survey and interviews with local seed retailers

In parallel with the sampling of vegetative material, farmers in 26 households were interviewed (16 women, 7 men and 3 couples) about their agricultural practices with special emphasis on seed selection, recycling and exchange between farmers. Unfortunately, we were not able to correlate households interviewed with leaf-samples from the field and only in part from gardens (7 out of 14 households, see S1 Table). For the household seed samples, 19 out of 20 samples could be correlated to a household interview (see S2 Table).

Respondents were asked 58 questions concerning farming and maize seed management. The farm households sampled represented 25% of the 105 farming households in the village. The survey was done shortly after the start of the 2012/2013 maize growing season. Questions were therefore asked about the previous 2011/2012 growing season in order to cover all questions (i.e. related to conditions and circumstances around planting, growing conditions and harvesting). Nine of the 26 interviewed farmers had planted their particular maize field during the 2011–2012 growing season, while all of them indicated that they had planted maize in their garden during that season. Farmers commonly did not refer to the official varietal names for the maize provided to them through the formal seed system. They mainly distinguished purchased varieties only with regard to colour of the ear and sometimes using familiar varietal names such as ‘Silver King’ for white maize varieties (despite Silver King being a variety no longer in production). Local maize was commonly referred to as ‘Xhosa maize’ and maize was commonly divided into red (actually more yellow in colour) and white maize. Some farmers had a more detailed categorization of local maize varieties.

In addition two local agricultural supply stores were visited. Seed retailers were asked about OPVs and hybrid non-GM and GM maize seeds in stock at the time of sampling, and questions were asked to determine their knowledge on the special features and management practices of the GM seed they sold.

### DNA extraction and quality analysis

DNA –extractions were performed according to the protocol described in the NucleoSpin II Plant kit (Macherey-Nagel, Germany). Leaf samples (circa 1 cm^2^) were crushed by vortexing together with 3–6 glass beads (6 mm in diameter) in a 15 mL tube that had been chilled in liquid nitrogen for 5–10 minutes. Approximately 0.1 mL of the ground leaf material was then transferred to a 1.5 mL tube and DNA extraction was performed accordingly, where the 10 minute incubation at 65°C with lysis buffer and RNase A was increased to 1 hour.

DNA concentration and quality were checked using the Nanodrop1000 spectrophotometer (Thermo Scientific, USA). DNA concentrations and purity varied somewhat between samples, and we set a quality threshold at an OD260/280 ratio = 1.6. Samples with ratios below 1.6 were excluded from the study (n = 96), leaving a total of 796 leaf samples for further analysis.

Each seed sample was weighed, and ground up to coarse flour using a blender. The equipment was thoroughly washed and sterilized before each grinding. Approximately 1 mL of the flour was then used for DNA extraction which was performed according to the Macherey-Nagel NucleoSpin II Plant kit protocol, with the following changes: 1) homogenization of sample was optimized by adding 6–8 steel beads (1.3 mm in diameter) and 200 µL lysis buffer before shaking the sample for 3x 40 sec in a Fast Prep machine (Qbiogene, Canada) at velocity 6, after which additional 200 µL of lysis buffer was added before further processing, and 2) the 10 minute 65°C incubation of the sample, with lysis buffer and RNase A, was increased to 1 hour.

### Polymerase chain reactions (PCRs) and agarose gel electrophoresis

A working concentration of 25 ng/µL was made from each DNA sample before PCR by dilution in in deionized nuclease-free water (Ambion, USA). All PCRs were performed as according to the mastermix recommendations, using recommended annealing temperatures (depending on primers) and elongation times (depending on product size).

The maize leaf DNA samples were pooled (due to the large amount of samples) to sets of 10 leaves before the PCR and run in duplicates. The pooling was performed by mixing 1 µL (25 ng) DNA from each sample into 10-sample pools (total of 250 ng DNA). First, a primer for the detection of *p35s* (AF434709, GenBank) was used [25]. The PCR reactions were performed in a total volume of 25 µl containing 12.5 µL Dynazyme II Mastermix (Finnzymes, Finland), 1.5 µL nuclease-free H_2_O (Ambion, Life Technologies, USA) and 0.5 µL of each of the forward and reverse primer (final concentration 0.5 µM). Amplicons from the PCR were run on a 2.5% agarose gel (Lonza, Switzerland), using GelRed (Biotium, USA), at 90V for approximately 1.5 h. The amplicons were visualized in UV light using GelDoc (BioRad, USA). A ΦX174 Hae III Digest ladder (New England Biolabs, USA) was used for size determination.

The one leaf DNA pool that was positive for transgenes (*p35s*) in the initial PCR reaction was split up and re-analysed as individual samples, using the same primer-pair, in order to identify the specific sample(s) that were positive. Here, 100 ng DNA template (4 µL) was used in each reaction with 12.5 µL Dynazyme II, 7.5 µL nuclease-free H_2_O and 0.5 µL of each primer (final concentration 0.5 µM). Plant material identity and amplifiability was also confirmed by performing PCRs using primers for the housekeeping gene *zein* [26]. In order to identify the transgenic maize event, PCRs were run using gene specific and event specific primer pairs for the insect-tolerant MON810 (Cry1Ab [25] and VM [27], [28], respectively) gene cassette, and an event specific primer pair for the herbicide-resistant NK603 (forward primer MON810 1–5 [29] and reverse primer NK603 1–3 [30]) gene cassette, with only minor changes to the PCR programs.

Seed samples were initially screened running real-time PCR with P35S primers (TaqMan GMO Maize 35S detection kit, Applied Biosystems, USA) on a StepOnePlus real-time PCR machine (Applied Biosystems, USA). The results can be found in S1 Real-time PCR Data. The five samples that showed positive results were tested further using end-point PCR. PCRs were run using primer pairs p35s, Cry1Ab, Cry1Ab2 (construct specific, GenØk, based on AF434709, GenBank) and VM, directed at detecting insect-resistance traits, and primer pair MON810 1–5/NK603 1–3 directed at detecting the herbicide-resistant event NK603. The PCR reactions were performed as follows: 12.5 µL Dynazyme II, 7.5 µL nuclease-free H_2_O, 0.5 µL of each primer (final concentration 0.5 µM), 4 µL (100 ng) DNA template, with only minor changes to the PCR programs. Gel electrophoresis of amplicons was done as described above, except for the PCR performed with primers Cry1Ab2, where a 0.8% gel was run due to size of the PCR product. The gel was run for 2 hours before visualization under UV light (GelDoc). A 1 kb ladder (New England Biolabs, USA) was used for size determination.

## Results

### Survey data on seed management

**Figure 2:**
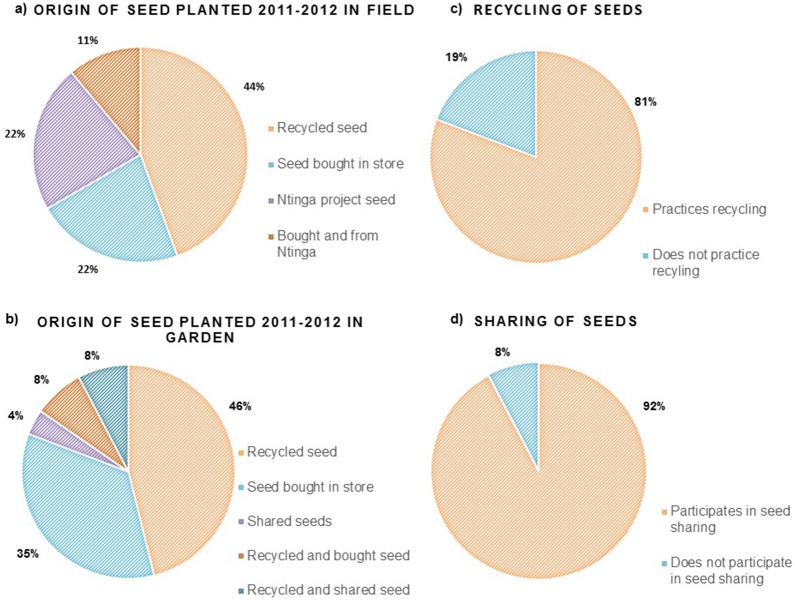
Survey Data. Survey data on seed origin showing what kind of seed was planted in a) fields and b) gardens during the 2011–2012 season; and on seed management in general showing the proportions of farmers c) recycling seed and d) participating in sharing (d). N = 26.

A high proportion of recycled seed was planted in gardens and fields in the 2011/2012 season (Fig. 2a and b). Some farmers combined seed from different sources, for example they supplemented their recycled seed with bought or shared seed and planted this together in their field or garden. Survey data shows that recycling of seeds in general (not limited to one particular year) was done by more than 80% of the farmers (Fig. 2c). Sharing of seeds (giving away or receiving seeds) was found to be very common and 92% reported to have participated in this activity at some time (Fig. 2d). Some would share seed every year, and others less often. Sharing would usually occur within the village or with farmers in other villages in the immediate vicinity. A few farmers also indicated that they gave seed to or received seed from people living more than 10 kilometres away. One farmer had received seed from a farmer approximately 700 km away.

When the respondents were asked if some types of maize were better for recycling than others, 73% of them answered *yes*, 8% answered *no*, and 19% were not sure or did not answer the question. Of the respondents saying that some maize types were better for recycling than others, 84% were of the opinion that local maize varieties were better suited, and 79% claimed that shop bought or project maize (maize supplied by agricultural projects) were worse for recycling. Often these two opinions went hand in hand. Notably however some farmers claimed that you could find maize in the shops that in essence behaved in the same way as the local varieties. Our interpretation of this, based on previous studies in the area [4], [5], is i) that smallholders have very little knowledge about seeds from the formal system (as they in essence group all seed from retailers and agricultural projects together) and ii) that there is much exchange between local seed pools and the locally popular improved OPVs purchased through the formal seed system.

Retailers confirmed that most smallholder in the region purchase OPV maize. The main reason for this was the price difference, hybrid seed being approximately three times as expensive as OPVs, and GM seed costing five times more than OPVs. Many farmers rarely asked for a specific variety of maize but only asked for white or yellow maize. Our interviews with retailers showed that shop attendants had very limited knowledge on the seed they were selling. We see a connection between this and the smallholders’ limited knowledge about the formal seed system and management practices. Retailers for example confirmed that farmers asking for the locally popular white OPV ‘Silver King’ were given other white OPVs as Silver King is no longer in production. This often without telling farmers that they were actually not purchasing ‘Silver King’. We also witnessed how local seed retailers repacked certified GM maize containing stacked traits for insect resistance and herbicide tolerance and relabelled the new bags only with the herbicide tolerant trait. It is common practice for seed retailers in the region to repack maize seeds into smaller 2 kg and 5 kg bags to cater for smallholders with a limited budget. Interviews with retailers also confirmed that shop attendants were unaware of the technology licencing agreement associated with GM seed. As a result smallholders were not asked to sign any technology licensing agreement when purchasing GM seed, which means that there was no formalized way of making sure that the farmers were given information about the specific management requirements surrounding GM crops. Such licencing agreements are mandatory when seed of GM crops are purchased in South Africa, both as a user agreement between the seed developer and the farmer, but also because it is part of the environmental release permit conditions.

### Detection of transgenes in plant material

**Figure 3:**
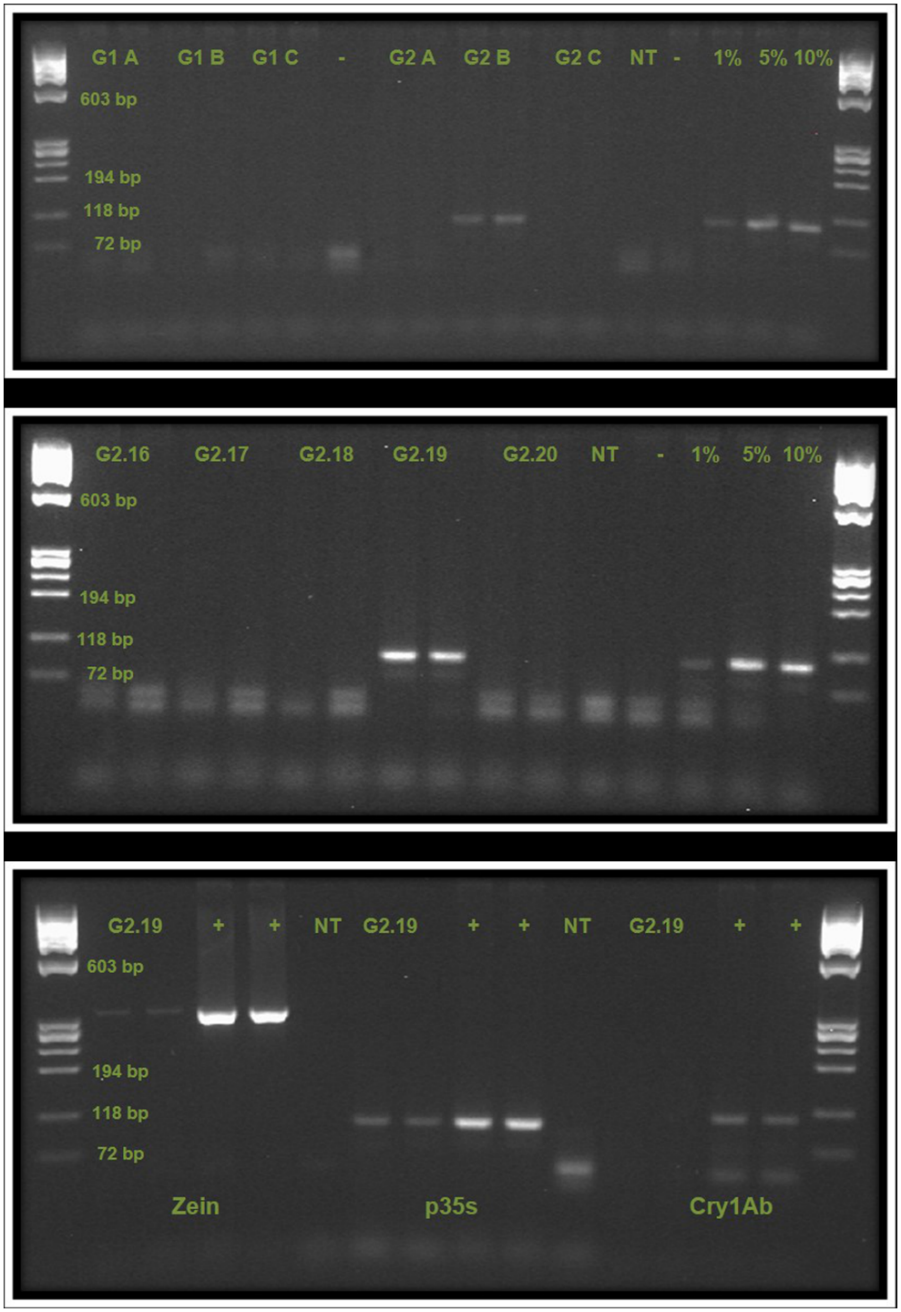
PCR detection of *p35s* in pooled and individual samples of DNA from maize leafs, and further tests of positive garden sample. Top Gel Pooled samples from sample sets G1 (A, B, C), and G2 (A, B, C), in duplicate, each pool consisting of 10 individual samples. Controls are no template (NT), negative (−), and 1%, 5% and 10%. G2 B is positive for the presence of *p35s*. Middle Gel Individual leaf DNA samples from G2 B pool, G2.16-G2.20, in duplicate. Controls are NT, negative (−) and positive controls (1%, 5% and 10%). G2.19 is positive for the presence of *p35s*. Bottom Gel Sample G2.19 tested for the presence of (left to right) *zein*, *p35s*, and *Cry1Ab* (in duplicates). Control used were positive (+) and NT. G2.19 is positive for *zein* and *p35s*. Ladder is ØX174 RF Hae III in all cases.

For the maize leaves collected in the field, only one sample (G2.19) was found positive for the *p35s* gene indicating the presence of a transgene insert (Fig. 3). We were unable to further identify the event due to the poor DNA quality of later DNA extracts from the leaf, probably due to the degraded condition of the leaf after storage and transport.

### Maize seed

**Figure 4:**
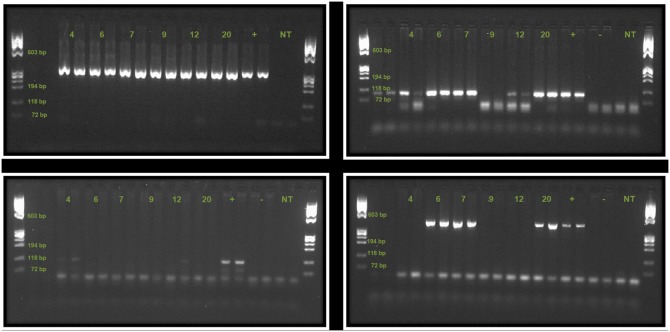
PCR results from maize seed samples. Top Left Gel Detection of the reference gene *zein* (duplicate samples), controls are positive (+) and no template control (NT). All samples are positive. Top Right Gel Detection of the transgene *p35s* (duplicate samples), controls are positive (+), negative (−) and no template control (NT). Samples 4, 6, 7, 12 and 20 are positive. Bottom Left Gel Detection of *Cry1Ab* (insect resistance gene, duplicate samples), controls are positive (+), negative (−), and no template control (NT). Sample 4 is positive, while sample 12 only shows a positive in one of the two lanes. Bottom Right Gel Detection of NK603 (herbicide tolerant event, duplicate samples), controls are positive (+), negative (−) and no template control (NT). Samples 6, 7, and 20 are positive. Ladder is ØX174 RF Hae III. A summary of these results can be found in Table 1.

**Figure 5:**
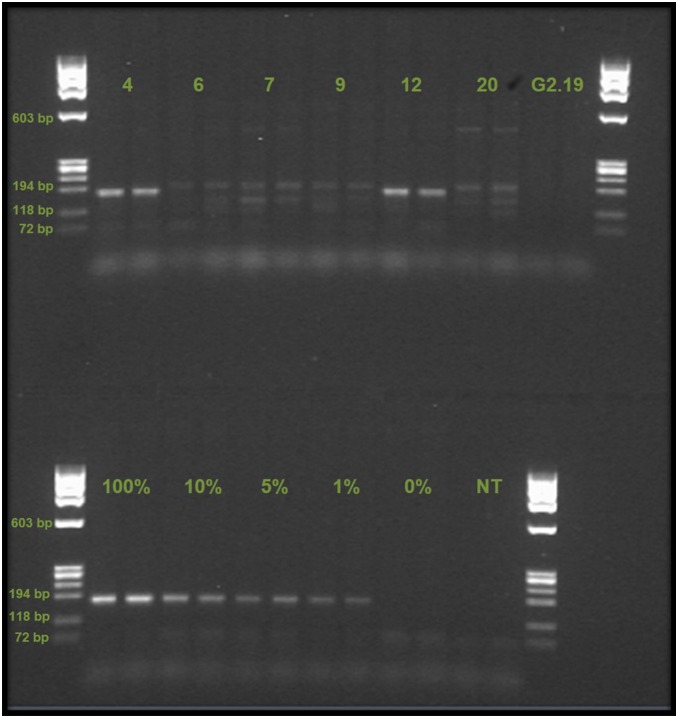
PCR results from reaction with MON810 event specific primers VM. Upper section of gel shows seed samples (4, 6, 7, 9, 12, and 20) and the one leaf sample (G2.19) that was further investigated. Lower section shows controls consisting of a gradient (100%, 10%, 5%, 1% and 0% MON810 content), and a no template control (NT). Only samples 4 and 12 showed clear signals at the correct position in the gel (VM product size 170 bp). Ladder used was ØX174 RF Hae III.

Of the 20 seed samples five (25%) amplified positively for the *p35s* gene (samples 4, 6, 7, 12 and 20) both with real-time (results supplied in S1 Real-time PCR Data) and end-point PCR (Fig. 4). These samples were further analysed by end-point PCR (LOD 1%, see Fig. 5). Seed sample 1 also showed signs of amplification in the real-time PCR, but very late in the reaction (near C_T_40) and close to the background noise, and was excluded from further testing.

Two samples (number 4 and 12) contained the MON810 event (see Fig. 4 for PCR results with Cry1Ab specific primers, and Fig. 5 for result with MON810 event specific primers VM). Sample 4 was a locally recycled variety, claimed to be Xhosa White maize, while sample 12 was bought in a store and perceived by the farmer to be the locally popular OPV “Silver King”. On the origin of the latter the farmer told us that she had purchased seed both in the local supermarket and from the seed retailer. She showed us a packet labelled ‘Silver King’ which makes it unlikely that she purchased at the seed retailers (who had confirmed that Silver King was no longer available). Three samples (number 6, 7 and 20) contained the NK603 event (Fig. 4, Bottom Right). Farmer interviews confirmed that seed samples 6 and 20 originated from herbicide tolerant seed bought intentionally, while sample 7 consisted of seeds originating from the Ntinga projects herbicide tolerant maize. Both farmers were aware of the maize’s herbicide tolerance, however when asked about this the first farmer said that he thought the Bt-maize would be resistant to Roundup as well. The other farmer said she believed other ‘strong’ maize varieties such as Silver King would tolerate ‘chemicals’ just as well as the ‘project’ maize. Whether or not these farmers use Roundup on their crops later in the season is not known. However, one aim of the Ntinga project was to help farmers treat their Roundup Ready crops with Roundup.

When running PCR with the MON810 event specific primer pair VM we found that while seed sample 4 and 12 were clearly positive, several of the other samples showed many weak smaller and larger PCR signals. For a summary of the PCR results on detection of transgenes, including information on sample origin and farmers participation in seed recycling and sharing, see Table 1.

**Table 1:** Summary of PCR results.

Sample no.	Real-time PCR p35s	PCR zein	PCR p35s	PCR Cry1Ab	PCR MON810	PCR NK603	Origin of seed	Farmer recycled seed?	Farmer sharing of seed
G2.19^†^	N/A	+	+	n.d.	n.d.	n.d.	Local (recycled 2 yrs.)	n.d.	n.d.
4	+	+	+	+	+	−	Local	Yes	Received seed
6*	+	+	+	−	−	+	Shop (Roundup Ready)	Rarely	Shared seed
7	+	+	+	−	−	+	Ntinga Project (Roundup Ready)	Yes	Both shared and received seed
12	+	+	+	n.d.	+	−	Shop	No	Shared seed
20*	+	+	+	−	−	+	Shop (Roundup Ready)	Rarely	Shared seed

PCR result summary for samples in which transgenes were detected, and background information on these samples. A positive result for Cry1Ab or MON810 indicates that the maize has an insect resistant trait, and a positive result for the NK603 indicates herbicide tolerance. Last two columns are based on the farmer surveys. N/A not applicable, N. d. = not determined, ^†^Sample G2.19 is a leaf from a maize plant growing in a garden, the remaining samples are seed samples, *Samples 6 and 20 are from the same farmer.

## Discussion

This study illustrates how transgenes and GM crop plants may enter into South African smallholder’s agricultural practices and seed storages beyond the farmers’ own knowledge. Only one leaf sample out of 796 (0.0013%) tested positive for a transgene (the *p35s* promoter, sample G2.19). This leaf sample originated from a garden. The farmer planting this garden told us that the seeds came from maize that she had recycled for two seasons and that she originally bought it in town, as ‘white maize’. As GM seed is significantly more expensive than the commonly purchased OPV maize, we conclude that it is highly unlikely that this woman originally bought a certified GM maize variety. We find it likely that she either purchased non-GM seeds that were contaminated with transgenes two years ago, or that her non-GM seeds had cross-hybridized with locally grown GM maize during the last two growing seasons.

Seed sample 4 which tested positive for *cry1Ab* and the MON810 event (insect tolerant) originated from recycled seed which the farmer considered to be a local variety. This farmer did not purchase seed in town but had recycled her own seed for a number of years and on occasion asked friends and neighbours for seed. The farmer was not aware that she had planted or stored GM maize.

Seed sample 12 tested positive for the MON810 event, but the presence of the *cry1Ab* gene could only be confirmed in one of the PCR duplicates (Fig. 4, bottom left), the same occurred when performing new PCRs with the sample. This ambiguous result (a positive signal for the MON810 event, implies the presence of the *cry1Ab* gene) most likely reflects low quality of the DNA or a very low initial concentration of the transgene in the DNA sample. According to the farmer this seed was bought in town. The farmer had asked in the shop for the locally popular, but no longer sold, OPV ‘Silver King’ and was of the belief that she planted this variety. The farmer was unaware that her maize contained transgenes.

In both examples discussed above the PCRs gave relatively weak signals compared to the controls (that contain 100% GM maize). From the strength of the signals, the estimated transgene content of these seed samples falls between 1 to 5% (see gradient in Fig. 5). These two seed samples, found to contain the *cry1Ab* transgene, confirmed by positive results for the MON810 event specific PCR, indicate that the MON810 event has become part of the locally recycled maize. However, the original source of the transgenes is unclear. The last report of certified MON810 being planted in this village stems from 2008. The MON810 positive samples observed in this study could either stem from MON810 supplied through the agricultural programme and planted up to 2008 (and recycled since then), or it could be the result of intermingling of GM and non-GM seeds or plants at the level of seed producers, seed retailers or between farmers. In addition, it is possible to buy maize seeds intended for food or feed use in local markets and shops, which are still viable and may contain GM material. With regards to the unspecific bands observed in the MON810 event specific PCR (primer pair VM) we are at this stage unable to provide an explanation, but it warrants further investigation.

The two farmers that provided the material testing positive for the event NK603 (n = 3 samples) had bought herbicide tolerant hybrid seeds at local retailers or received the seeds from Ntinga OR Tambo Development Agency. These farmers cultivated GM maize knowingly: one because she had left-over seeds from the Ntinga project, and the other because he wanted to further test this new seed beyond the timeframe of the Ntinga project. Neither of the two farmers seemed to be aware of the specific link between NK603 as herbicide tolerant maize and the herbicide Roundup, and none of them had signed a technology licensing agreement when buying or receiving these seeds. This is a repeat of what has been reported from the past development intervention run by the Massive Food Production Program where it has been established that farmers likewise planted insect tolerant maize without signing technology licensing agreements [4]. Given the close proximity of fields and gardens in the village, high rates of gene flow by pollen transfer must be expected between GM and non-GM varieties if both types of maize are planted [31], [32]. A high rate of seed exchange was also demonstrated as more than 90% of the interviewed local farmers shared seeds with other farmers from time to time. We could confirm that all farmers with positive test results for transgenes both recycled seeds and participated in sharing of seeds.

Our investigations have revealed how locally recycled maize varieties may be intermingled with GM maize through several different pathways. One possible pathway is the planting of GM seeds from agricultural programs (or seed retailers) in close proximity to non-GM seed, even in the same fields, where mixing can occur through pollen flow. Another pathway is through the seed retailers that often have nonspecific, unclear and occasionally incorrect information on the seed they stock. A third possibility is the sale and use of uncertified seeds. In the studied village, practices of seed recycling and sharing seed were common, and although farmers may not deliberately buy GM seeds they may still plant, recycle and/or share these unknowingly, i.e. beyond their and the permit holders control and without adhering to the biosafety conditions indicated in the approval and licencing of GM crops (such as records of plant locations, management plans and strategies).

Resistance of the African maize stem borer, *Busseola fusca* (Lepidoptera: Noctuidae) to Bt-toxin was first reported in South Africa during 2006 [33]. Resistance has increased in range and intensity over the years [34], [35]. The main reasons behind this resistance development were found to be low compliance to refugia planting among commercial farmers, combined with late and variable planting times and high infestation levels [36]. The introduction and spread of Bt-transgenes in crops in smallholder communities may contribute to the development of resistance; in the event of cross pollination between insect tolerant plants and local varieties a new generation of seeds with variable and sometimes reduced expression levels of Bt-toxin will be expected [37]. It is recognized that exposure to low levels of the Bt- toxin could accelerate the development of resistance in target insects [37]–[39]. However, the overall distribution of GM and non-GM plants within the area and the local population dynamics of the target insect will also influence the rate at which resistance develops [40], [41].

Smallholders commonly intercrop maize with other food plants such as pumpkin and beans [5]. Growing herbicide tolerant maize and spraying with broad-spectrum glyphosate-based herbicides would make such intercropping difficult. However, less tillage would be necessary [42]). Spraying of herbicides in small-scale farming could potentially also interfere with the crops grown on surrounding fields through herbicide drift [43], [44].

In smallholder maize farming communities, farmers knowingly buying GM seed because of recommendation, or because of its novelty, might not know what GM-seeds actually are. Assefa and Van den Berg [15] and Jacobson and Myhr [4] have reported from different South African smallholder communities that farmers were not properly informed about traits and management practices of GM-maize and that they often adopted it for reasons not relevant or unique to GM maize, for example, high yield, drought tolerance, fast growth and good taste. Jacobson [5] found that there was a common understanding (in the village investigated in the present study) that neither Bt maize nor herbicide tolerant maize could be intercropped. Facilitating good and precise communication between the farmers and the agricultural projects is challenging. For example smallholders have reportedly treated stem borer resistant crops with chemicals targeted at stem borers, and reduced chemical treatments against pests to which the GM crop is not resistant [16], [45], [46].

In our interviews, we confirmed that many farmers were not informed about the permit conditions surrounding the use of GM seed when they bought or received the seeds. This is in line what has been reported by Jacobson and Myhr [4] who found that farmers were unaware that it was mandatory to plant refugia when planting Bt-maize and that they did not know that it is illegal to share almost any certified seed (including OPVs). Similar findings are reported from other parts of the world as well. Stone [47], [48] has reported from communities in India where retailers and farmers both displayed insufficient or incorrect knowledge about the pest resistance trait of Bt-cotton and its respective management requirements. The main reason for this lack of knowledge was absence of state sponsored, independent, agricultural advice, forcing farmers and retailers to depend on commercial seed promotion for information. A similar situation has been identified in South Africa [4], [5], [15]. Indeed the frequent inability amongst retailers to supply smallholders with adequate information on agricultural products is acknowledged to be a major problem across countries in the global South [49]. There are also reports of smallholders consciously ignoring information given regarding management of GM crops, particularly those regarding recycling and sharing of seed as these practices are widespread and deep-rooted in most smallholder communities. An earlier study from the village in the present study reported that the farmers in the region, despite confirming that they had been informed about not being allowed to recycle GM seed, frequently ignored this and continued recycling seed [4]. Jacobson [5] argues that the South African patent legislation, plant breeders’ rights and biosafety management practices are in fact incompatible with the smallholder practices of recycling and sharing seed, and that this could undermine strategies for ensuring food security.

Non-compliance to management practices, e.g. to limit resistance development is however not only an issue amongst smallholders. Kruger, van Rensburg and Van den Berg [34], [36] showed that non-compliance to the use of refuges was high amongst large-scale farmers. There are stewardship programs directed at commercial farming systems in South Africa, and on-farm inspections are used to investigate cases of possible non-compliance [34]. However, this system is not suitable for the smallholders because most of these farmers will not be in any registers or have access to the right kind of information (as exemplified by the lack licencing contracts and the lack of knowledge among seed retailers).

The sampling design of the study covered leaf material both from fields and home gardens, as well as seeds stored in houses. However, the coverage of the total number of fields and farmers was nevertheless limited in our study. In addition there might be a sampling bias because only farmers who were present at the time of our visits were asked to participate. Full overlap between sampled gardens/fields and interviews would have provided better knowledge on the extent to which farmers’ responses on varietal names and origin of seed corresponded with genetic analysis.

Although the data material presented here has some limitations, we argue that our findings present important information about effects of introducing GM crops in smallholder communities. As discussed, findings presented here support findings made in smallholder communities in other regions. We suggest that further follow-up studies, drawing on larger and more comprehensive data, are made to improve on the representativeness of our findings and provide further insight into the dynamics of the smallholder system in South Africa.

Theoretical studies (modelling) of smallholder communities in Ghana and Zambia indicate that the combination of pollen flow between fields, and extensive recycling/sharing of seeds, would lead to uncontrolled spread and persistence of transgenes, if GM maize plants were introduced. [10]–[12]. This study highlights the problems that occur when transferring stewardship strategies and management measures for GM crops (e.g. the use of refugia and segregation of GM and non-GM plants) adapted to large-scale farming into small-scale agriculture.

## Conclusion

This study shows that transgenes are present in non-GM maize varieties in a South African smallholder community. Although the data is limited and only from a single village in Eastern Cape, we show that: i) GM seed is bought or recycled and grown outside the formal seed sector; ii) GM maize is grown in such close proximity to other locally recycled and purchased maize varieties that transgene flow must be expected at a high rate; iii) transgenes from MON810 were found four years after the last certified MON810 crop was grown in the village; iv) seed and information from local retailers are often of dubious quality so that locally purchased seed may represent a vector for contaminated maize seed. The combination of these factors ultimately results in unintended mixing of GM and non-GM maize germplasm. This has potential ecological consequences, such as resistance development, as well as possible legal consequences with infringement of IPRs and permit conditions. Our results point at the difficulties in cultivating GM and non-GM crops together in rural smallholder subsistence farming communities under current permit regulations for GM crops.

## Supporting Information

S1 Table **Overview of all garden sample sets, each sample set representing one garden and consisting of one leaf from 30 individual maize plants.** The column ‘Maize variety’ gives the name that the farmers provided us with during the collection of the samples. The column ‘Origin of maize’ contains the information we were given on the origin of the plants, in cases were the maize was recycled we tried to get information on where the original seeds came from, however this proved difficult. Under PCR results, the p35s column indicates how many of the 30 leafs in the sample test were positive for p35s. Then follows a test for the maize reference gene *zein*, the insect resistant trait Cry1Ab, and the insect resistant event MON810 (Bt-maize). Lastly a test for the herbicide tolerant event NK603 (Roundup Ready) is given. Positive and negative results are indicated by + and −, respectively. Finally, information is given on whether the farmer participates in recycling or sharing of seeds (in the cases where this information was accessible.
(PDF)

S2 Table **Overview of all seed samples, each sample consisting of between 58 to 119 seeds, donated by a farmer.** The maize variety and the origin of the seeds as far as we were able to track it with the farmers is given in columns ‘Maize variety’ and ‘Origin of maize’. The weight of the seed samples and estimated number of seeds are given in the next two columns. Under ‘PCR results’ the first column indicates which samples were positive of the *p35s* transgene. Then follows a test for the maize reference gene *zein*. Two tests for the presence of insect resistance genes were performed, and results are shown in the columns ‘Cry1Ab’ for the presence of the *cry1Ab* transgene, and in column ‘MON810’ (Bt-maize) for the event specific tests. The results of the test for the NK603 event (Roundup Ready) is shown in the last column under ‘PCR results’. Finally, information is given on whether the farmer participates in recycling or sharing of seeds (in the cases where this was given).
(PDF)

S1 Real-time PCR Data **Data from the real-time PCR reaction initially run on the seed samples in order to screen for the presence of the** ***p35s*** **transgene.** Using the TaqMan GMO 35S Detection kit (Applied Biosystems, USA), on a StepOne Plus real-time PCR machine (Applied Biosystems, USA) the reporter molecule was FAM and the quencher molecule NFQ-MGB. C_t_ threshold was set to 0.10201. As the results show, samples 4, 6, 7, 12 and 20 clearly amplified indicating the presence of *p35s*. Sample 1 also began to amplify, but very late the reaction (after 38 cycles), and its results were very similar to background noise, and thus sample 1 was considered to be negative for *p35s*. Since this was run with qualitative purposes, quantification based on standard curves was not performed. Also includes a linear amplification plot with the positive samples 4, 6, 7, 12, and 20 marked by blue arrows, and the excluded sample 1.
(PDF)
