# Supplementary material for: Detection of Transgenes in Local Maize Varieties of Small-Scale Farmers in Eastern Cape, South Africa
Source: PLoS One. 2014 Dec 31;9(12):e116147. doi: 10.1371/journal.pone.0116147 (PMC4281112; doi:10.1371/journal.pone.0116147)
Supplement: S1 Table — Overview of all garden sample sets, each sample set representing one garden and consisting of one leaf from 30 individual maize plants. The column ‘Maize variety’ gives the name that the farmers provided us with during the collection of the samples. The column ‘Origin of maize’ contains the information we were given on the origin of the plants, in cases were the maize was recycled we tried to get information on where the original seeds came from, however this proved difficult. Under PCR results, the p35s column indicates how many of the 30 leafs in the sample test were positive for p35s. Then follows a test for the maize reference gene zein, the insect resistant trait Cry1Ab, and the insect resistant event MON810 (Bt-maize). Lastly a test for the herbicide tolerant event NK603 (Roundup Ready) is given. Positive and negative results are indicated by + and −, respectively. Finally, information is given on whether the farmer participates in recycling or sharing of seeds (in the cases where this information was accessible. (PDF) [file pone.0116147.s001.pdf]

Supplementary Information S1

Gardens	Maize variety	Origin of maize	PCR results					Farmer activities		Comments
			p35s	zein	Cry1Ab	MON810	NK603	Recycles	Shares	
G1	Xhosa White Maize	n.d.	0	n/a	n/a	n/a	n/a	n.d.	n.d.	
G2	White Maize	Recycled	1	+	-	-	-	Recycles	n.d.	Issues with DNA quality of 2nd and 3rd DNA extraction, probalby due to the poor condition of the leaf after transport and storage.
G3	Xhosa White Maize	n.d.	0	n/a	n/a	n/a	n/a	n.d.	n.d.	
G4	Xhosa White Maize	Recycled	0	n/a	n/a	n/a	n/a	Recycles	Shares	
G5	Xhosa White Maize (Silver King)	Recycled	0	n/a	n/a	n/a	n/a	Recycles	Shares	
G6	Silver King and Red Maize	Recycled	0	n/a	n/a	n/a	n/a	Recycles	Shares	Planted together in garden
G7	Red and White (local Silver King) Maize	Red maize is bought in the Agricultural Shop, while the White maize is recycled	0	n/a	n/a	n/a	n/a	Recycles	Shares	Planted together in garden
G8	Xhosa White	n.d.	0	n/a	n/a	n/a	n/a	n.d.	n.d.	
G9	McDonalds "Border King"	Agricultural Shop	0	n/a	n/a	n/a	n/a	No	Shares	Same household as sample G10
G10	Xhosa Red	Recycled	0	n/a	n/a	n/a	n/a	No	Shares	Same household as sample G9
G11	Silver King (White Maize) and Xhosa Red	Recycled	0	n/a	n/a	n/a	n/a	Recycles	Shares	
G12	Xhosa White Maize	n.d.	0	n/a	n/a	n/a	n/a	n.d.	n.d.	
G13	Red Maize	n.d.	0	n/a	n/a	n/a	n/a	n.d.	n.d.	
G14	White Maize	Bought in the Agricultural Shop	0	n/a	n/a	n/a	n/a	n.d.	n.d.	

Supplementary Information S1

Table 1 Garden Samples Overview of all garden sample sets, each sample set representing one garden and consisting of one leaf from 30 individual maize plants. The column 'Maize variety' gives the name that the farmers provided us with during the collection of the samples. The column 'Origin of maize' contains the information we were given on the origin of the plants, in cases were the maize was recycled we tried to get information on where the original seeds came from, however this proved difficult. Under PCR results, the p35s column indicates how many of the 30 leafs in the sample test were positive for p35s. Then follows a test for the maize reference gene *zein*, the insect resistant trait Cry1Ab, and the insect resistant event MON810 (Bt-maize). Lastly a test for the herbicide tolerant event NK603 (Roundup Ready) is given. Positive and negative results are indicated by + and -, respectively. Finally, information is given on whether the farmer participates in recycling or sharing of seeds (in the cases where this information was accessible.
