# Supplementary material for: Detection of Transgenes in Local Maize Varieties of Small-Scale Farmers in Eastern Cape, South Africa
Source: PLoS One. 2014 Dec 31;9(12):e116147. doi: 10.1371/journal.pone.0116147 (PMC4281112; doi:10.1371/journal.pone.0116147)
Supplement: S2 Table — Overview of all seed samples, each sample consisting of between 58 to 119 seeds, donated by a farmer. The maize variety and the origin of the seeds as far as we were able to track it with the farmers is given in columns ‘Maize variety’ and ‘Origin of maize’. The weight of the seed samples and estimated number of seeds are given in the next two columns. Under ‘PCR results’ the first column indicates which samples were positive of the p35s transgene. Then follows a test for the maize reference gene zein. Two tests for the presence of insect resistance genes were performed, and results are shown in the columns ‘Cry1Ab’ for the presence of the cry1Ab transgene, and in column ‘MON810’ (Bt-maize) for the event specific tests. The results of the test for the NK603 event (Roundup Ready) is shown in the last column under ‘PCR results’. Finally, information is given on whether the farmer participates in recycling or sharing of seeds (in the cases where this was given). (PDF) [file pone.0116147.s002.pdf]

## Supplementary Information S2

| Seed samples | Maize Variety | Origin of maize | Weight (g) | Estimated number of seeds | PCR results | | | | | Farmer activities | | Comments |
|---|---|---|---|---|---|---|---|---|---|---|---|---|
| | | | | | p35s | zein | Cry1Ab | MON810 | NK603 | Recycles | Shares | |
| 1 | Red Maize | Bought 2 yrs. ago in the Agricultural Shop, then recycled. | 30.4 | 101 | - | n/a | n/a | n/a | n/a | Recycles | Shares | Same household as sample 2 |
| 2 | Silver King | Bought 2 yrs. ago in the Agricultural Shop, then recycled. | 32 | 107 | - | n/a | n/a | n/a | n/a | Recycles | Shares | Same household as sample 1 |
| 3 | Xhosa White Maize | Recycled | 20.4 | 68 | - | n/a | n/a | n/a | n/a | Recycles | Shares | |
| 4 | Xhosa White Maize | Recycled | 25.8 | 86 | + | + | + | + | - | Recycles | Shares | |
| 5 | Silver King, Xhosa Maize | Recycled | 34.6 | 115 | - | n/a | n/a | n/a | n/a | Recycles | Shares | Same household as sample 15 |
| 6 | Roundup Ready (DKC78-35R) | Agricultural Shop | 32.8 | 109 | + | + | - | - | + | Recycles | Shares | Same household as sample 20. Recycles, but not often |
| 7 | Roundup Ready | Ntinga Project | 23.3 | 78 | + | + | - | - | + | Recycles | Shares | Same household as sample 10 and 11 |
| 8 | Xhosa Yellow Maize | Recycled | 31 | 103 | - | n/a | n/a | n/a | n/a | Recycles | Shares | |
| 9 | Local White Maize (Silver King), Red Maize | Recycled White Maize, Red maize from Agricultural Shop | 32.6 | 109 | - | n/a | n/a | n/a | n/a | Recycles | Shares | Mixed seeds together |
| 10 | Project maize | Recycled for two years | 31.9 | 106 | - | n/a | n/a | n/a | n/a | Recycles | Shares | Same household as sample 7 and 11, might be derived from NK603 from the Ntinga project. |
| 11 | Silver King | Recycled | 29.9 | 100 | - | n/a | n/a | n/a | n/a | Recycles | Shares | Same household as sample 7 and 10 |
| 12 | Silver King | Agricultural Shop | 34.9 | 116 | + | + | **n.d.** | + | - | No | Shares | |
| 13 | Xhosa Red Maize | Recycled | 30.5 | 102 | - | n/a | n/a | n/a | n/a | Recycles | Shares | Same household as sample 5 |

## Supplementary Information S2

| | | | | | | | | | | | | | |
|---|---|---|---|---|---|---|---|---|---|---|---|---|---|
| 14 | White Maize | Received from relatives outside the village | 27.4 | 91 | **-** | n/a | n/a | n/a | n/a | Recycles | Shares | |
| 15 | McDonalds "Mac Medium Pearl" | Agricultural Shop | 33 | 110 | **-** | n/a | n/a | n/a | n/a | Recycles | Shares | |
| 16 | CHEEKY White Maize | Grocery Shop | 17.5 | 58 | **-** | n/a | n/a | n/a | n/a | Recycles | Shares | Recycles, but not the last few years |
| 17 | Silver King/Xhosa White Maize | Recycled | 32 | 107 | **-** | n/a | n/a | n/a | n/a | Recycles | Shares | |
| 18 | Xhosa Maize, mixed seeds (Black Maize, Silver King/White Maize, Red Maize) | n.d. | 34.5 | 115 | **-** | n/a | n/a | n/a | n/a | n.d. | n.d. | Likely recycled seed |
| 19 | Xhosa Maize (Silver King) | Recycled | 21.8 | 73 | **-** | n/a | n/a | n/a | n/a | Recycles | Shares | Farmer told us this variety had white kernels on a red cob. |
| 20 | Roundup Ready | Recycled | 35.6 | 119 | **+** | **+** | **-** | **-** | **+** | Recycles | Shares | A whole cob, same household as sample 6. Recycles, but not often. |

**Table 1 Seed Samples** Overview of all seed samples, each sample consisting of between 58 to 119 seeds, donated by a farmer. The maize variety and the origin of the seeds as far as we were able to track it with the farmers is given in columns 'Maize variety' and 'Origin of maize'. The weight of the seed samples and estimated number of seeds are given in the next two columns. Under 'PCR results' the first column indicates which samples were positive of the *p35s* transgene. Then follows a test for the maize reference gene *zein*. Two tests for the presence of insect resistance genes were performed, and results are shown in the columns 'Cry1Ab' for the presence of the *cry1Ab* transgene, and in column 'MON810' (Bt-maize) for the event specific tests. The results of the test for the NK603 event (Roundup Ready) is shown in the last column under 'PCR results'. Finally, information is given on whether the farmer participates in recycling or sharing of seeds (in the cases where this information was given).
