# Supplementary material for: Detection of Transgenes in Local Maize Varieties of Small-Scale Farmers in Eastern Cape, South Africa
Source: PLoS One. 2014 Dec 31;9(12):e116147. doi: 10.1371/journal.pone.0116147 (PMC4281112; doi:10.1371/journal.pone.0116147)
Supplement: S1 Real-time PCR Data — Data from the real-time PCR reaction initially run on the seed samples in order to screen for the presence of the p35s transgene. Using the TaqMan GMO 35S Detection kit (Applied Biosystems, USA), on a StepOne Plus real-time PCR machine (Applied Biosystems, USA) the reporter molecule was FAM and the quencher molecule NFQ-MGB. Ct threshold was set to 0.10201. As the results show, samples 4, 6, 7, 12 and 20 clearly amplified indicating the presence of p35s. Sample 1 also began to amplify, but very late the reaction (after 38 cycles), and its results were very similar to background noise, and thus sample 1 was considered to be negative for p35s. Since this was run with qualitative purposes, quantification based on standard curves was not performed. Also includes a linear amplification plot with the positive samples 4, 6, 7, 12, and 20 marked by blue arrows, and the excluded sample 1. (PDF) [file pone.0116147.s003.pdf]

Supplementary Information S3

Sample Name	Task	Cт	Cт Mean	Cт SD
No template control	NTC	Undetermined		
No template control	NTC	Undetermined		
No template control	NTC	39.54932		
Sample 1	UNKNOWN	38.27925	38.05571	0.27541
Sample 1	UNKNOWN	37.74805	38.05571	0.27541
Sample 1	UNKNOWN	38.13981	38.05571	0.27541
Sample 2	UNKNOWN			
Sample 2	UNKNOWN			
Sample 2	UNKNOWN	Undetermined		
Sample 3	UNKNOWN	Undetermined	37.03977	
Sample 3	UNKNOWN	Undetermined	37.03977	
Sample 3	UNKNOWN	37.03977	37.03977	
Sample 4	UNKNOWN	33.67116	33.39337	0.246241
Sample 4	UNKNOWN	33.30697	33.39337	0.246241
Sample 4	UNKNOWN	33.20197	33.39337	0.246241
Sample 5	UNKNOWN	Undetermined		
Sample 5	UNKNOWN	Undetermined		
Sample 5	UNKNOWN	Undetermined		
Sample 6	UNKNOWN	26.44509	26.45942	0.032622
Sample 6	UNKNOWN	26.43641	26.45942	0.032622
Sample 6	UNKNOWN	26.49675	26.45942	0.032622
Sample 7	UNKNOWN	26.99835	27.08795	0.077983
Sample 7	UNKNOWN	27.12498	27.08795	0.077983
Sample 7	UNKNOWN	27.14052	27.08795	0.077983
Sample 8	UNKNOWN	37.79433	37.79433	
Sample 8	UNKNOWN	Undetermined	37.79433	
Sample 8	UNKNOWN	Undetermined	37.79433	
Sample 9	UNKNOWN	Undetermined		
Sample 9	UNKNOWN	Undetermined		

Supplementary Information S3

Sample 9	UNKNOWN	Undetermined		
Sample 10	UNKNOWN	Undetermined		
Sample 10	UNKNOWN	Undetermined		
Sample 10	UNKNOWN	Undetermined		
Sample 11	UNKNOWN	Undetermined		
Sample 11	UNKNOWN	Undetermined		
Sample 11	UNKNOWN	Undetermined		
Sample 12	UNKNOWN	33.34803	33.12369	0.228045
Sample 12	UNKNOWN	33.13093	33.12369	0.228045
Sample 12	UNKNOWN	32.89211	33.12369	0.228045
Sample 13	UNKNOWN	Undetermined		
Sample 13	UNKNOWN	Undetermined		
Sample 13	UNKNOWN	Undetermined		
Sample 14	UNKNOWN	Undetermined		
Sample 14	UNKNOWN	Undetermined		
Sample 14	UNKNOWN	Undetermined		
Sample 15	UNKNOWN	Undetermined		
Sample 15	UNKNOWN	Undetermined		
Sample 15	UNKNOWN	Undetermined		
Sample 16	UNKNOWN	Undetermined		
Sample 16	UNKNOWN	Undetermined		
Sample 16	UNKNOWN	Undetermined		
Sample 17	UNKNOWN	Undetermined		
Sample 17	UNKNOWN	Undetermined		
Sample 17	UNKNOWN	Undetermined		
Sample 18	UNKNOWN	Undetermined		
Sample 18	UNKNOWN	Undetermined		
Sample 18	UNKNOWN	Undetermined		
Sample 19	UNKNOWN	Undetermined		
Sample 19	UNKNOWN	Undetermined		
Sample 19	UNKNOWN	Undetermined		
Sample 20	UNKNOWN	28.79233	28.6517	0.123428

Supplementary Information S3

Sample 20	UNKNOWN	28.56135	28.6517	0.123428
Sample 20	UNKNOWN	28.60141	28.6517	0.123428
Positive control	+	34.14617	32.90111	2.092847
Positive control	+	34.0723	32.90111	2.092847
Positive control	+	30.48488	32.90111	2.092847
Negative control	-	Undetermined		
Negative control	-	Undetermined		
Negative control	-	Undetermined		

Table 1 Real-time PCR Results Data from the real-time PCR reaction initally run on the seed samples in order to screen for the presence of the *p35s* transgene. Using the TaqMan GMO 35S Detection kit (Applied Biosystems, USA), on a StepOne Plus real-time PCR machine (Applied Biosystems, USA) the reporter molecule was FAM and the quencher molecule NFQ-MGB. C_t threshold was set to 0.10201. As the results show, samples 4, 6, 7, 12 and 20 clearly amplified indicating the presence of *p35s*. Sample 1 also began to amplify, but very late the reaction (after 38 cycles), and its results were very similar to background noise, and thus sample 1 was considered to be negative for *p35s*. Since this was run with qualitative purposes, quantification based on standard curves was not performed. Also see figure 1 below.

Supplementary Information S3

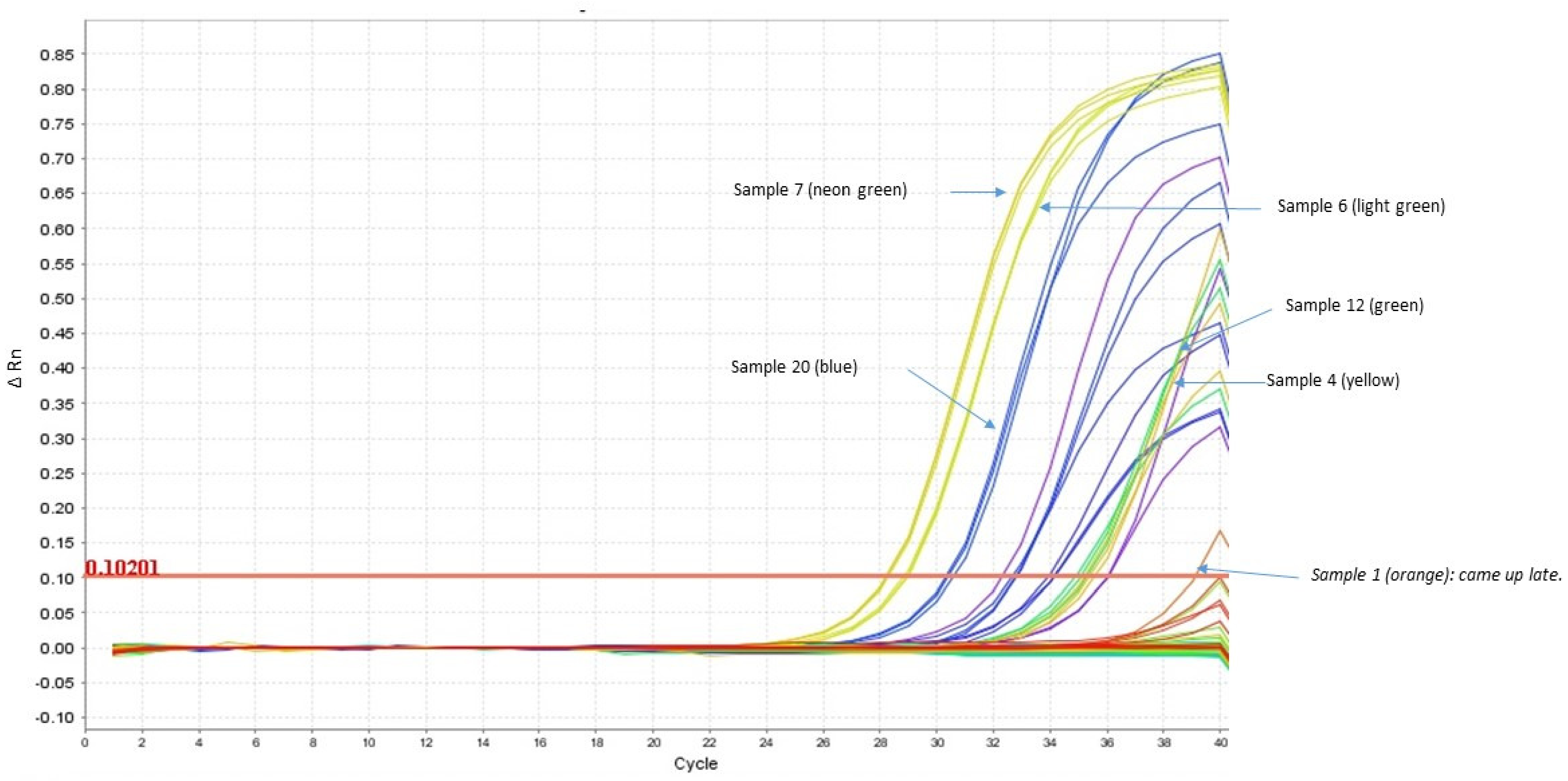

Figure 1 Linear amplification plot with the positive samples 4, 6, 7, 12 and 20 marked by blue arrows, and the excluded sample 1 also marked by a blue arrow.

Supplementary Information S3
